# Artificial Intelligence and Assistive Robotics in Healthcare Services: Applications in Silver Care

**DOI:** 10.3390/ijerph22050781

**Published:** 2025-05-14

**Authors:** Giovanni Luca Masala, Ioanna Giorgi

**Affiliations:** School of Computing, University of Kent, Canterbury CT2 7NZ, UK; i.giorgi@kent.ac.uk

**Keywords:** AI in healthcare, aged care, elderly care, assistive robotics, social robotics

## Abstract

Artificial intelligence (AI) and assistive robotics can transform older-person care by offering new, personalised solutions for an ageing population. This paper outlines recent advances in AI-driven applications and robotic assistance in silver care, emphasising their role in improved healthcare services, quality of life and ageing-in-place and alleviating pressure on healthcare systems. Advances in machine learning, natural language processing and computer vision have enabled more accurate early diagnosis, targeted treatment plans and robust remote monitoring for elderly patients. These innovations support continuous health tracking and timely interventions to improve patient outcomes and extend home-based care. In addition, AI-powered assistive robots with advanced motion control and adaptive response mechanisms are studied to support physical and cognitive health. Among these, companion robots, often enhanced with emotional AI, have shown potential in reducing loneliness and increasing connectedness. The combined goal of these technologies is to offer holistic patient-centred care, which preserves the autonomy and dignity of our seniors. This paper also touches on the technical and ethical challenges of integrating AI/robotics into eldercare, like privacy and accessibility, and alludes to future directions on optimising AI-human interaction, expanding preventive healthcare applications and creating an effective, ethical framework for eldercare in the digital age.

## 1. Introduction

Societies globally are getting older at a pace much faster than in the past. The figures indicate that individuals aged 60 have already outnumbered children younger than 5, with this demographic projected to surpass 1.4 billion by 2030 [1]. While longer lives present exciting opportunities, new challenges are posed for healthcare systems to deliver quality, accessible and sustainable care for older adults [2]. Ageing affects individuals differently, and thus, we need to fully understand the priorities of older adults and genuinely recognise their contributions to society. The key issues revolve around providing education and resources for long-term conditions [3]. Health systems in place for older individuals feel fragmented, reactive and disconnected, often creating inequity and ageism and leaving both patients and providers unhappy [4,5]. Moreover, the availability of paid and unpaid caregivers is dwindling, leading to overburdened carers [6], a rationing of health services and unmet needs for the elderly [7].

This demographic shift and the associated public health challenges have accelerated the research in advanced technologies in healthcare, especially artificial intelligence (AI) and robotics, to support eldercare [8]. AI and robotics can step up to address the perennial shortage of healthcare workers in many countries and ease the burden on professionals and family carers. Increasing expert voices argue that “*medicine could be on the brink of an AI revolution … and change the way care is delivered moving forward*” [9]. Furthermore, wearables or smart devices, often powered by AI, offer complementary solutions that are adaptable outside the clinical environment, like in care homes or at home [10].

This paper offers a perspective on the promise of technological innovations to improve or fill gaps in eldercare by examining current advances in AI, assistive robotics and hybrid systems while acknowledging associated ongoing risks and limitations.

## 2. The Role of Artificial Intelligence in Healthcare Services

The AI jargon in healthcare typically refers to computer systems that use maths, logic and patterns learned from the data to achieve human intelligence, reasoning, decision making and providing recommendations [11]. The past decade has seen strong interest in using artificial intelligence (AI) to improve diagnostics, e.g., in radiology [12]. Currently, healthcare systems like the NHS are increasingly using AI for clinical and administrative support, both in primary care and hospitals, for example, to reduce no-shows or short-notice cancellations, optimise patient triage, personalise treatment plans, improve wound care and cataract care. [11]. Machine learning and deep learning (DL), which are the current state of the art in AI, have demonstrated tremendous progress in image analysis and have established a benchmark method for image classification and recognition. Machine learning typically extracts essential features from images, whereas DL utilises deep neural networks to do the same with superior accuracy [13]. Both methods have been consistently used to detect diseases on the skin, liver and heart and other medical conditions that need to be diagnosed early [14]. In addition to diagnosis, AI is often used for various operational tasks in the radiology workflow [15]. There are many AI applications in development at present, showing great promise across each phase of the imaging process, from ordering tests to delivering reports [16]. These advantages have significant implications for addressing the specific needs of older-person care, like the early diagnosis of age-related diseases, e.g., age-related macular degeneration [17], Parkinson’s disease [18] and the diagnosis of Alzheimer’s disease [19].

A major open problem in eldercare is designing AI solutions that can predict health deterioration, optimise medication management and assist individuals and their carers with rudimentary daily tasks. Machine learning and natural language processing are being explored to detect subtle changes in the physical and cognitive health of elderly patients, which enables early intervention and reduces hospital readmissions. At present, AI and robotics solutions are promising numerous benefits. To illustrate, AI-driven predictive analysis allows healthcare professionals to identify risks early, create personalised treatment plans and take preventive actions. For example, AI algorithms can process large datasets and recognise patterns in the data that may indicate early-stage dementia [20] or the likelihood of falls [21]. This proactive approach allows caregivers to make data-informed decisions, resulting in better health outcomes for older adults. In addition, integrating these solutions into wearable devices [22] can enhance elderly care by monitoring health, detecting emergencies and providing targeted assistance.

## 3. Robotics in Elderly Care

With its dual role in physical and emotional support, robotics is a major part of the growing recognition that silver care must be holistic to address not only medical needs but also the social and emotional aspects of health. Assistive robotics in older-person care is thus bipartite, and commonly sub-categorised into service robotics and social robotics [23]. Service robots are intended to enhance ease of living by performing practical non-industrial functions safely, such as cleaning, mobility support and item delivery. Social robots are designed to engage with humans in rudimentary conversations, providing companionship, cognitive stimulation and emotional support [23]. With that said, typical current advances in assistive robotics go beyond the programming of simple rules and reactive behaviour and necessitate sophistication with AI models for navigation, planning, vision recognition, predictive maintenance, natural language processing and emotion recognition, to mention a few, into sophisticated hybrid systems [24]. With proper design, assistive robots can enhance the quality of life for elderly individuals and help them maintain a degree of independence, which is essential for preserving their dignity and self-worth [8].

Service robots present exciting potential for the elderly. A well-established deployment of service robots to support aged care is in the preparation and dispensing of medicines, which typically resides within pharmacies and often involves pharmacy automation, robotic systems and human supervision [25]. In recent years, these have moved from pharmacies to homes and care homes through the commercialisation of automatic dispensers like the Philips Medication Dispenser and Black & Decker Pria [26]. The latter stands out for its hybrid nature as an automatic dispenser and social robot, providing interactions through facial recognition and voice assistance, as well as telepresence with caregivers. This telepresence forms part of a broader shift towards remote aged care, in particular, reaching home-dwelling individuals living alone or in underserved locations. In such cases, falls pose a serious threat, where significant incident rates with potential fatal outcomes are prominent among those aged 60 or older. Thus, growing research efforts have been placed on the early prevention of falls using AI, commonly deep learning models, with IoT (Internet of Things) technology to provide round-the-clock monitoring in real time, early detection, alerts and timely emergency response [27].

Service robots can also support elderly patients with limited mobility. There have been initial studies to help patients with assistive dressing [28] and bathing assistive devices [29], with prototypes like Robear and HUG [30] promising to transform nursing care, but they have yet to be normalised. These applications are more difficult to realise, given constraints on human–machine interactions and the specificity of the patient’s physiological condition [31], particularly for fragile older individuals. However, recent years have seen significant developments for use in physiotherapy and rehabilitation, with high precision and versatility in delivering consistent motion exercises, with examples in post-stroke rehabilitation [32], upper limb rehabilitation [33] and robot-assisted surgery [34], applicable and adaptable to older adults. However, this level of service robotics is not ready yet. The tasks are complex for the current state of the art, and their solutions may not have been fully customised or tested for the elderly, making safe interactions with people and the environment still a technological challenge.

Therefore, social robotics has emerged as a promising and widely explored alternative in care settings for older adults, offering companionship and cognitive stimulation. Social robots are often designed as intelligent companions, which are interactive, AI-powered machines designed to provide emotional support, companionship and assistance to users. They often display high anthropomorphism and mimic human or animal behaviours, respond to voice commands and adapt to user preferences. Older adults are increasingly becoming familiar with robot pets, particularly through the use of affordable commercial robotic animals in care homes, such as robotic cat toys for the elderly affected by dementia. Relevant research in the last two decades on human–robot interaction with older adults has revealed optimistic results from petting animal-like robots, with similar effects as in therapies with animate pets in improving pain and lowering anxiety, blood pressure and neuropsychiatric symptoms (e.g., [35]). Examples of currently available products are the seal-shaped Paro [36]; the dog-shaped and cat-shaped JfA [37], AIBO [38]; and the dinosaur-shaped Pleo [39]. Robot pets are easier to maintain since they do not require feeding, cleaning or routine care tasks, unlike real animals. Additionally, they have fewer restrictions regarding living conditions, for example, in apartments, hospitals or assisted living facilities where real animals may not be practical. They are also programmed to behave consistently and predictably, which can be reassuring for individuals with cognitive impairments.

Of late, more advanced companion robots are emerging as an alternative to companion animals. To develop companion robots that owners genuinely cherish, it is crucial to understand the depth of attachment they form with their robot [40]. Some recent, more artful explorations of emotional relationships between humans and robots address this idea. For example, the work of Takayuki Todo, “Dynamics of a Dog on a Chain”, features robotic dogs chained to pillars, evoking complex emotional responses from viewers and sparking discussions about freedom, control and human–machine bonds. When it comes to such effects for the elderly, social robots, more or less humanoids, use conversational and emotional AI to engage elderly users in meaningful interactions [41], often alleviating loneliness [42] and improving mental health outcomes [43]. Popular solutions include Pepper [44], NAO [45], Ryan [46], ElliQ [47], Buddy Pro [48], iPal [49] and Care-O-Bot 4 [50]. In addition to improving connectivity, robots (and AI virtual agents/chatbots) also promote cognitive health by providing brain-stimulating games and exercises, including for patients with mild cognitive impairment (MCI) [51]. These features help users stay mentally engaged, support cognitive function and lower the risk of decline linked to isolation.

In addition to the clinical and operational benefits, recent applications are exploring AI and robotics in commensality [52], with social robots participating in shared mealtimes, enhancing social presence and fostering positive reactions. The idea of eating with a robot, either in place of or alongside other people, provides a novel approach to combating loneliness and promoting engagement. However, while the social presence of robots during mealtimes has shown promise, there is yet to be significant evidence that it impacts food intake. Despite this, such innovations highlight the broader potential of AI and robotics to enrich the lives of older adults beyond medical care alone.

When designing robots for therapeutic use with older adults, we often focus on assessing their trust and acceptance of the technology. It is, however, equally important to consider their input in the design process and what they really want from robots. Older adults do not want to be seen as a problem and are willing to contribute to co-designing solutions. For example, the study in [53] highlights the importance of stakeholder engagement, participatory design, and user-centred approaches in AI healthcare solutions, emphasising how integrating a social robotic system into nursing care can enhance quality while actively involving older adults in the design process. As the technology advances, robots that can help individuals beyond their current features in daily tasks like bathing, dressing and feeding will become readily available. Instead of undermining human touch, they will help our seniors preserve their independence and alleviate onerous tasks and physical strain for human carers.

## 4. Trust in Robots for Sensitive Tasks

**Figure 1:**
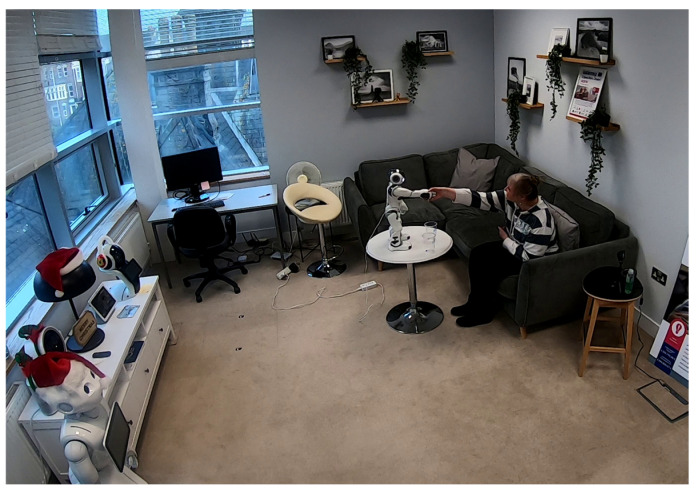
Non-functional robot touch (handshake) experiments with older person(s) to measure their trust during medicine administration [55].

Recent research on trustworthy robotics has moved beyond just understanding the causes and effects of trust in robots and is now focused on how robots can gain, calibrate and maintain the trust of their human users [54]. Human–robot interaction (HRI) in sensitive tasks, especially in eldercare and healthcare, is a complex dynamic involving trust, reliability and the task itself. Focused studies highlight the key factors that influence how older adults perceive and interact with robots in these contexts and offer valuable insights into the challenges and opportunities of integrating robotic systems into sensitive domains (e.g., dispensing medication). The authors of [55] showed that trust in robots is dependent on both their social agency (i.e., perceived warmth) and their ability to perform tasks reliably (Figure 1). Indeed, the authors found that while older adults liked a robot that exhibited social behaviours such as benevolence and touch, these attributes did not mitigate the impact of errors in critical tasks like medication administration. Trust was significantly reduced when the robot made an error (experimental condition), regardless of its humanlike qualities. Thus, in sensitive tasks, reliability is the primary driver of trust, trumping anthropomorphism or social engagement. The study also found that trust in robots correlates with older adults’ willingness to adopt them in their homes, especially for health-related applications. However, much remains to be seen if humanlike interactions of robots are enough to satisfy deeper emotional and social needs.

**Figure 2:**
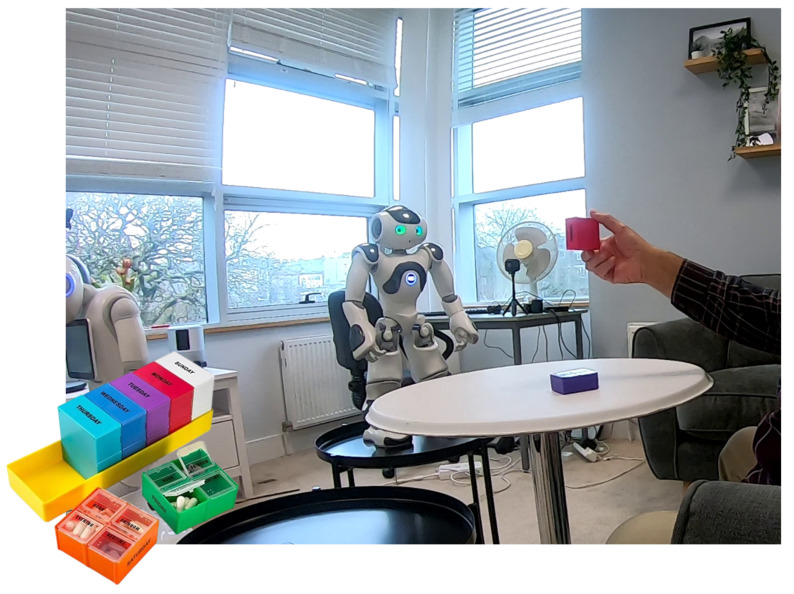
An older person interacting with a robot to study trust in robot-provided drug advice, examining preferences for robot information-based versus recommendation-based guidance [56].

The authors of [56] expanded on the discussion by exploring the role of robots in providing health-related advice (Figure 2). Instead of focusing on specific attributes such as anthropomorphism or competence, the research examined whether older adults preferred information-based advice over recommendation-based advice on mild drugs. Contrary to expectations, trust in the robot remained stable regardless of the type of advice it provided, suggesting that older adults were generally receptive to robot-based guidance in low-risk health contexts. However, the study also indicated that task complexity plays a role in shaping trust. When participants received information-based advice from the robot on supplements, they trusted the robot less for advice on medicines. Instead, when the robot provided recommendation-based advice, i.e., it suggested supplements instead of simply describing them, older adults continued to trust its advice in also recommending medicines for more severe conditions. This finding suggests that offering recommendations rather than just factual information may enhance the perception of a robot’s expertise. Both studies in [55,56] underscore that trust in robots is neither static nor universal but is heavily context-dependent. In low-risk scenarios, such as supplement recommendations, older adults were open to receiving advice from robots, demonstrating a moderate level of trust before and after interaction. However, when the stakes were higher, as in medication administration, the consequences of robotic reliability became more pronounced, leading to fluctuating trust. This highlights a critical consideration in designing robotic systems for sensitive tasks: while social attributes can enhance engagement, they cannot compensate for perceived unreliability in high-risk situations. Moreover, the findings suggest that the labelling of a robot as an expert by a human professional may positively influence people’s trust and compliance levels, though further investigation is needed to determine the extent of this effect. The implications for future research include exploring the nuances of trust transfer between low-risk and high-risk tasks, assessing the role of different robot characteristics (such as confidence and expertise), and conducting real-world studies to validate these insights in practical applications.

Ultimately, these studies reinforce findings that trust in robotic advisers and caregivers is a multifaceted issue. Further empirical research is needed to establish best practices for integrating robots into eldercare and healthcare environments effectively.

## 5. Challenges of Implementing AI and Robotics in Elderly Care

### 5.1. General Challenges

While AI and robotics promise to ease the burden of (elderly) care, implementing these technologies is a challenge. One of the primary hurdles is the value proposition [57]. The high price of advanced AI systems and assistive robots may be prohibitive for some healthcare facilities, especially smaller institutions and those in low-income areas. Additionally, the cost of maintaining and updating these technologies can strain healthcare sectors on already stretched budgets. However, despite being prohibitively expensive, current social robots come with significant technical limitations. They tend to generate disruptive noise and often struggle with speech recognition, especially when dealing with regional accents, excessive background noise or users with speech impairments, which is a common issue among older adults. Moreover, they often suffer from limited battery life, motor overheating or inconsistent connectivity [57]. In some circumstances, these limitations may go beyond just user experience to potentially compromise safety. Issues like malfunctions, misrecognitions, the hallucinations of AI algorithms, like giving contradictory medical advice or failing to detect falls, or even potential physical harm from both service and social robots, like tripping on a robot hoover [58], can be more dangerous for elderly individuals due to their increased vulnerability [59].

Another critical challenge is the need for the extensive training of carers. Caregivers must operate AI systems and robotic devices effectively, requiring both time and resources, which may be met with resistance [60]. Already under time and emotional pressures, they may see these innovations as yet another responsibility, requiring new training, unfamiliar routines and adjustments to workflows [61]. Such training must also be provided with no assumptions about previous staff experience and digital literacy, and no practice gap between robotic engineering and healthcare settings [62].

Furthermore, older persons themselves may struggle to adapt to technologies, especially if they include complex interfaces or if the individuals have limited digital literacy or physical dexterity. This can lead to frustration and resistance associated with usability issues, posing non-trivial acceptability and adoption barriers [63]. As with carers, bridging any gaps between AI systems and end-users is essential to ensuring these technologies serve their intended purpose. For this purpose to be achieved, equitable access must inform the design and deployment of the technology in ways that remove social, geographical, economic and cognitive barriers, so that all older adults can benefit regardless of their circumstances [64]. Therefore, for such technologies to be truly effective and embraced, implementation must go hand in hand with thoughtful integration, inclusive design and a clear demonstration of long-term benefits, both for the care recipients and for those delivering the care, through high-quality studies and randomised controlled trials [23,57].

Data privacy and security are also predominant subjects in the adoption of AI in healthcare [65]. AI systems rely on a vast amount of personal data to make accurate predictions and provide personalised care. The recent use of large language models (LLMs), while revolutionary, is further exacerbating the problem of data ownership and trustworthiness [66]. Ensuring that these data are securely stored and processed while protecting patient privacy is a challenging yet essential aspect of ethical AI use in healthcare. The potential for data breaches or the misuse of sensitive information is a risk that healthcare providers must address through robust cybersecurity measures and adherence to strict data protection regulations [67].

Despite their conceptual allure, robots must overcome significant usability and accessibility hurdles before they can be meaningfully integrated into everyday care settings.

### 5.2. Ethical Considerations

When it comes to elderly care, ethics are key to introducing AI and robotics. Using robots to assist or supplement human care raises questions about what care is and the importance of the human touch. While robots can meet some physical needs, they cannot be emotionally attuned companions like human carers. This risks “dehumanising” elderly care as patients feel reduced to interacting with machines rather than people. The unintended dehumanising effects of AI are not just from biases but also from achieving goals flawlessly [68]. This may raise a risk of dependence or overreliance as older adults, especially those whose reasoning may be impaired, may be more vulnerable to trusting automated systems uncritically [59]. Autonomy and consent are thus significant ethical issues, especially for older people with cognitive impairments. We must ensure that AI and robotic systems are used in a way that respects the autonomy of older patients and involves them in their care. Older people have diverse and specific needs and limitations that must be considered in the human-centred design process [69]. A human-centred approach to AI and robotics in eldercare is key to ensuring technology enhances, not replaces, human dignity, empathy and personal care. By putting the needs, values and well-being of older adults first, we can create responsible and compassionate solutions that empower, not dehumanise, build trust and improve quality of life.

Transparency in AI decision-making is imperative. Patients and their families should understand how algorithms arrive at particular recommendations or predictions. Equally important is transparency in a robot’s design and behaviour. As robots become more advanced, they risk crossing an ethical line with deceitful anthropomorphism [70]. When a robot “closes its eyes” or turns away, a user might reasonably assume they are no longer being observed, so they might behave or talk more freely than they would if they knew they were being watched. In contexts like eldercare or therapy, where users are already in a vulnerable position, this illusion of privacy becomes not just misleading but potentially exploitative.

Lastly, we need ethical guidelines and policies to regulate the use of AI and robotics in healthcare. An “ideal” regulatory framework has yet to materialise. Uncertainties around data protection, privacy and the redefinition of healthcare relationships, such as the role of AI-based systems and robots in doctor–patient interactions, are left behind by current legal frameworks [59]. The fast-paced AI and evolving nature of care robots are challenging the regulatory category boundaries; for example, it is not clear whether they should fall under medical devices or consumer technology. As such, regulations are created ad hoc, duplicated or present gaps in oversight [59]. Thus, governments and healthcare organisations need to work together to create harmonised and standardised frameworks that put patient welfare first, ensure equitable access to new technology and address the biases in AI systems. The significant movement of the European Union to establish a common regulatory framework through the EU Artificial Intelligence Act (AI Act) [71,72], which addresses both material harm (safety and health) and immaterial harm (privacy loss, human dignity, discrimination and bias), represents a comprehensive effort in this direction. However, until its full provision, current AI systems and robotics applications may not be covered by any regulation, risking opaque decision-making, discriminatory outcomes, misuse or intrusions that require continuous attention and refinement of the legal framework at the same pace as technological progression.

Ethical design means clarity, not just in documentation but also in form and function. When users interact with machines that look and behave like they are sentient beings, their assumptions should be respected and guided, not betrayed.

## 6. Conclusions

The benefits of AI solutions and assistive robotics are numerous for the elderly and their carers. AI promises to rescue health and aged care from data and work overload. To accelerate diagnosis and personalise treatment, larger, complex patient data are needed, and AI can turn these data into accurate, actionable predictions at the right time and with greater speed and consistency than human analysis alone [9]. Moreover, assistive robotics can provide the care older adults need, offering this care where it is currently lacking due to a caregiver shortage or in underserved locations, or by taking some of this burden off overwhelmed professionals and families. The physical, cognitive and social support offered by these robots can improve the elderly’s quality of life, enable independent home or community-dwelling, mitigate loneliness and enhance connectedness, inclusion and autonomy for a holistic well-being. With ongoing development, effective deployment “in the wild” and user-centric design, when combined with wearable devices and smart systems, AI will help professionals make better decisions and achieve better outcomes for a more responsive, preventive approach to care for ageing societies.

While bumps and potholes exist, such as the necessity for high-quality, diverse and unbiased data to train AI algorithms; technical limitations; costs; user training; safety; and ethical concerns, including equity in access, accessibility and usability, the benefits of technology in eldercare cannot and must not be ignored. Careful and thoughtful implementation is needed to integrate AI/robotics and smart/wearable devices to transform eldercare, making it more efficient, sustainable and attuned to the needs of our seniors. A balanced approach is key to realising the full potential of technology, so these advancements are tools for all concerned rather than just substitutes for human interaction.

## Data Availability

This manuscript has no associated data.
